# Reconciling Longitudinal Naive T-Cell and TREC Dynamics during HIV-1 Infection

**DOI:** 10.1371/journal.pone.0152513

**Published:** 2016-03-24

**Authors:** Julia Drylewicz, Nienke Vrisekoop, Tendai Mugwagwa, Anne Bregje de Boer, Sigrid A. Otto, Mette D. Hazenberg, Kiki Tesselaar, Rob J. de Boer, José A. M. Borghans

**Affiliations:** 1 Laboratory of Translational Immunology, Department of Immunology, University Medical Center Utrecht, Utrecht, The Netherlands; 2 Theoretical Biology & Bioinformatics, Department of Biology, Utrecht University, Utrecht, The Netherlands; University of Pittsburgh Center for Vaccine Research, UNITED STATES

## Abstract

Naive T cells in untreated HIV-1 infected individuals have a reduced T-cell receptor excision circle (TREC) content. Previous mathematical models have suggested that this is due to increased naive T-cell division. It remains unclear, however, how reduced naive TREC contents can be reconciled with a gradual loss of naive T cells in HIV-1 infection. We performed longitudinal analyses in humans before and after HIV-1 seroconversion, and used a mathematical model to investigate which processes could explain the observed changes in naive T-cell numbers and TRECs during untreated HIV-1 disease progression. Both CD4^+^ and CD8^+^ naive T-cell TREC contents declined biphasically, with a rapid loss during the first year and a much slower loss during the chronic phase of infection. While naive CD8^+^ T-cell numbers hardly changed during follow-up, naive CD4^+^ T-cell counts continually declined. We show that a fine balance between increased T-cell division and loss in the peripheral naive T-cell pool can explain the observed short- and long-term changes in TRECs and naive T-cell numbers, especially if T-cell turnover during the acute phase is more increased than during the chronic phase of infection. Loss of thymic output, on the other hand, does not help to explain the biphasic loss of TRECs in HIV infection. The observed longitudinal changes in TRECs and naive T-cell numbers in HIV-infected individuals are most likely explained by a tight balance between increased T-cell division and death, suggesting that these changes are intrinsically linked in HIV infection.

## Introduction

Both CD4^+^ and CD8^+^ T-cell homeostasis are clearly disturbed during untreated HIV infection [1]: in the acute phase of infection, the majority of memory CD4^+^ T cells in the gut are lost [2,3] while in the chronic phase, peripheral CD4^+^ T cells are gradually lost. The CD8^+^ T-cell pool expands during the acute stage of infection and starts to decline at the AIDS stage [4], while the percentage of naive cells in the CD8^+^ T-cell pool is severely reduced throughout HIV infection [5–7]. The causes of these changes in the CD4^+^ and CD8^+^ T-cell pools are still debated.

HIV infection of the thymus, and a resulting decline in thymic output, has been suggested to contribute to the gradual loss of naive T cells in HIV infection [8–10]. In the absence of a direct measure of thymic output, T-cell receptor excision circles (TRECs) have been used to indirectly quantify how many cells are exported by the thymus per day [10]. TRECs are formed during V(D)J TCR gene rearrangement, and are not copied during cell division [11]. It has been shown that the average number of TRECs per T cell (referred to as “average TREC content”) declines with age in healthy individuals, and is significantly reduced in HIV-1 infected individuals [9,10,12,13]. Based on a mathematical model, it has previously been argued that the reduced average TREC content of T cells in HIV-1 infection is probably due to increased naive T-cell division, and provides no evidence for reduced thymic output [9].

Although increased naive T-cell division is indeed expected to lead to a reduction in the average TREC content, it is not clear how it can be reconciled with declining naive T-cell numbers. The increased naive T-cell loss that probably counteracts the effect of increased T-cell division on the size of the naive T-cell pool in HIV infection, is in fact expected to increase the average TREC content through "rejuvenation" of the T-cell pool [9,14], thereby also counteracting the TREC-diluting effect of increased T-cell division. The observed changes in the CD4^+^ and CD8^+^ T-cell pools during HIV infection are thus not trivially explained. Similarly, it remains unclear to what extent loss of thymic output can explain the changes in the T-cell pool during HIV infection, because naive T cells are very long-lived, with an average lifespan of 6–9 years in healthy individuals [15], and thymic output is responsible for only ~10% of daily naive T-cell production from the age of 20 years onward [16]. Our recent *in vivo* deuterium labeling study among treatment-naive HIV-1 infected individuals revealed that during chronic HIV-1 infection, naive T-cell production and loss rates are at least 3-fold increased, yielding life-expectancies of 1.7 and 0.7 years for CD4^+^ and CD8^+^ naive T cells, respectively [17]. With such quantitative insights at hand, it has become possible to study the expected changes in naive T-cell numbers and their TRECs during HIV-1 infection in the presence and absence of thymic impairment, and to study how reduced average TREC contents can be reconciled with declining naive T-cell numbers.

Here, we collected longitudinal data on naive T-cell numbers and TRECs over HIV-1 seroconversion and during the first five years of untreated HIV-1 infection, and used a mathematical model to study which factor(s) can explain their changes. In contrast to most previous studies, which followed the *percentage* of naive cells in the CD4^+^ and CD8^+^ T-cell pools during HIV infection [5–7], we also measured naive CD4^+^ and CD8^+^ T-cell *counts* during disease progression and over seroconversion. By studying TRECs and naive T-cell numbers longitudinally we circumvented the problems intrinsic to cross-sectional studies that are hampered by the large inter-individual differences in TREC levels and naive T-cell numbers. Indeed, a considerable overlap between healthy and HIV-infected individuals has repeatedly been reported [12,13,18,19]. We found that naive CD4^+^ and CD8^+^ TREC contents decrease biphasically, with a rapid decline during the first year and a much slower decline during the chronic phase of infection. Naive CD4^+^ T-cell numbers declined throughout infection, with no sign of a biphasic pattern, while naive CD8^+^ T-cell numbers remained stable during the 5-year follow-up. Using a mathematical model, we show that a combination of increased naive T-cell division and loss suffices to explain the biphasic TREC loss and the observed naive T-cell changes during HIV-1 disease progression, but only if the changes in T-cell division and loss are tightly linked. Thymic impairment, on the other hand, does not help to explain the observed biphasic TREC loss.

## Materials and Methods

### HIV- infected individuals

Samples from HIV-infected individuals for the longitudinal study (n = 18) were derived from the Amsterdam Cohort Studies (ACS) on HIV infection and AIDS. Cross-sectional data were derived from the ACS and from previous studies [9,20,21]. None of the HIV-infected individuals had been treated at the time of sampling. All sampling occurred between 1985 and 2005, a period in which treatment was either not yet available or typically only started below 350 CD4 T cells/μl. All individuals included in this study showed typical disease progression; long term non-progressors were not included. Individuals were considered acutely infected when sampling took place within 2 months after onset of symptoms of HIV infection (n = 7). We also included 27 HIV-infected patients in the chronic stage and 16 classified as AIDS. Data from age-matched blood bank donors were used as healthy controls (n = 38). Medical ethical approval from the University Medical Center Utrecht, the Amsterdam Medical Center and the ACS were obtained for this study and written informed consent was obtained from all participants.

### Flow cytometry and cell sorting

Peripheral blood mononuclear cells (PBMC) were obtained by Ficoll-Paque density gradient centrifugation from heparinized blood and viably frozen until further processed. Absolute CD4^+^ and CD8^+^ T-cell counts were determined by dual-platform flow cytometry. Effector/memory (CD27^+^CD45RA^-^, CD27^-^CD45RA^-^ and CD27^-^CD45RA^+^) and naive (CD27^+^CD45RA^+^) CD4^+^ and CD8^+^ T-cell fractions were assessed by flow cytometry as described previously and analyzed on a FACSCalibur with CellQuest software (Becton Dickinson (BD), San Jose, California) [22]. In 14 out of 32 healthy individuals, CD45RA^+^CD4^+^ T cells were positively selected by magnetic beads. Because CD45RA^+^CD27^-^ effector CD4^+^ T cells are virtually absent in healthy individuals (≤1.5% in 10 controls in whom we used magnetic beads and were able to measure the effector subset), this fraction represents CD45RA^+^CD27^+^ naive CD4^+^ T cells. To measure the TREC content within CD4^+^ and CD8^+^ T cells, these subsets were purified from thawed PBMC by positive selection using magnetic beads according to manufacturer’s instructions (Miltenyi Biotec Inc, Sunnyvale, California).

### TREC analysis

DNA was isolated using the QIAamp Blood Kit according to manufacturer’s instructions (Qiagen, Hilden, Germany). Signal joint (Sj) T-cell receptor excision circle (TREC) numbers were quantified using a real-time PCR method as previously described [9].

### Mathematical modeling of naive T-cell and TREC dynamics

Naive T-cell and TREC dynamics were investigated using a previously developed mathematical model [23], describing the changes in the total number of naive CD4^+^ T cells *N* and the total amount of TRECs *T* in the naive T-cell population, as a function of the time-dependent source of naive T cells from the thymus *σ(t)*, the average number of TRECs per recent thymic emigrant *c*, the rate *d* of naive T-cell loss through cell death and priming of naive into memory cells, and the rate of peripheral naive T-cell division *p*. More details about the model and the parameters are given in Supplemental Materials (S1 File).

### Statistics

A non-parametric Mann-Whitney test was performed for group comparisons. Differences between paired data during longitudinal follow-up were tested using Wilcoxon's signed ranks test. As pre-seroconversion values, we took the median of all T-cell counts that were available pre-seroconversion. Differences with a p-value < 0.05 were considered significant.

## Results

### Longitudinal follow-up of TRECs and T-cell numbers before and after HIV-1 seroconversion

We performed longitudinal analyses of the average TREC content of CD4^+^ and CD8^+^ T cells over HIV seroconversion in 18 treatment-naive typically progressing patients. The median time point at which samples were analyzed before these individuals got HIV infected was 4.8 years (range 2.8–11.4) before seroconversion. Since the expected TREC content decline in the period until seroconversion is negligible (with an average decline of 0.02 log_10_/year in healthy individuals [10,24]), we used everyone's pre-seroconversion TREC content as the best estimate of the TREC content at the time of seroconversion. TREC contents were also measured at a median of 1.0 year (range 0.8–1.3) and 5.0 years (range 3.5–5.2) post-seroconversion. Here forth, we refer to the period between seroconversion and 1 year post-seroconversion as phase I, and to the period between 1 and 5 years post-seroconversion as phase II. In addition to TRECs, absolute CD4^+^ and CD8^+^ T-cell counts (per μl of blood) and fractions of naive and effector/memory CD4^+^ and CD8^+^ T cells were determined.

**Fig 1:**
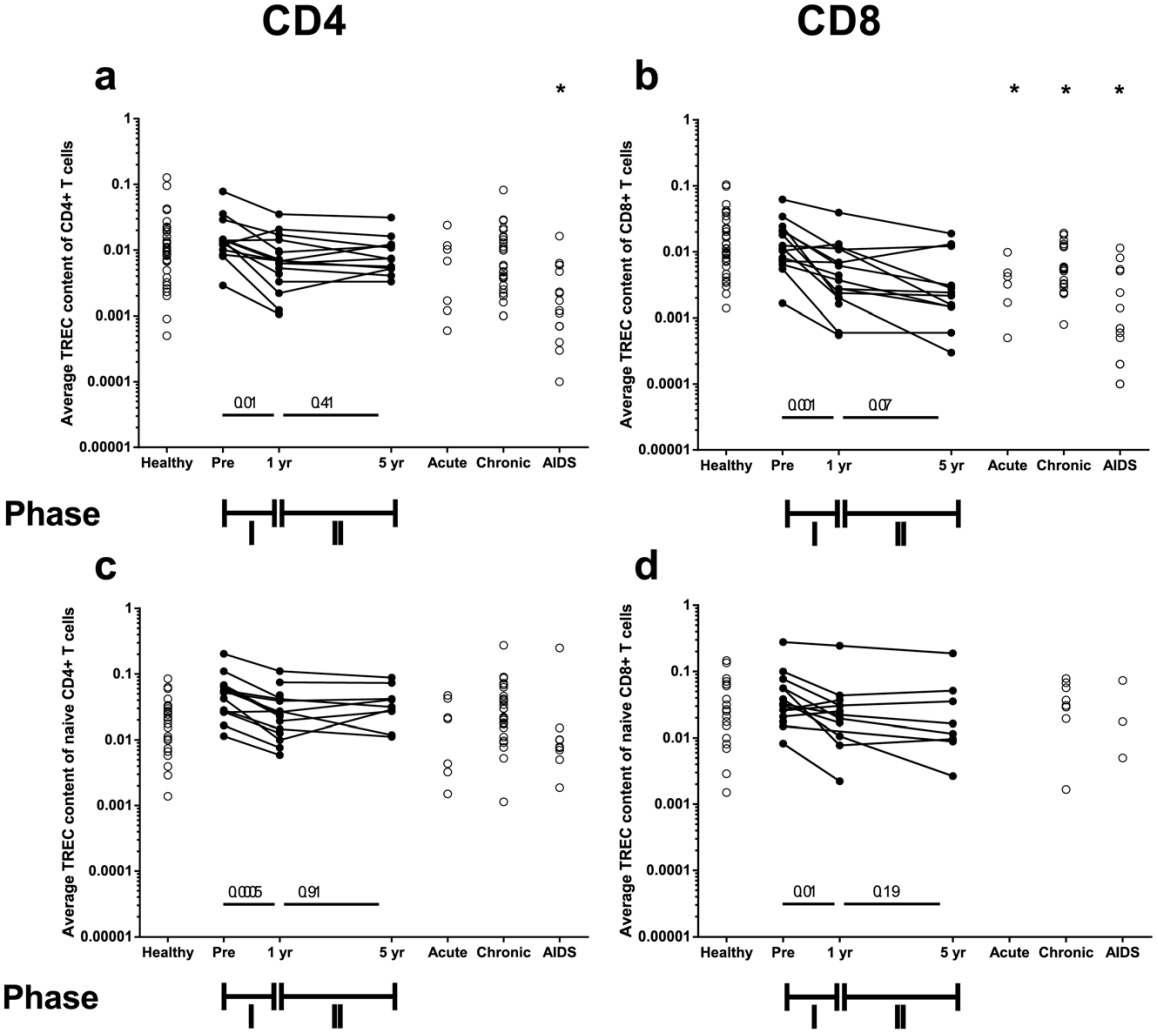
CD4^+^ and CD8^+^ TREC dynamics over seroconversion and during HIV infection. The average TREC contents for each individual in CD4^+^ T cells (a), CD8^+^ T cells (b), naive CD4^+^ T cells (c) and naive CD8^+^ T cells (d) measured over seroconversion and during HIV infection. The average naive TREC contents were calculated by dividing the average TREC content of each population by the fraction of naive cells in that population. Longitudinal data are connected by straight lines (n = 18), while cross-sectional data are denoted by open circles. The pre-seroconversion measurements (“Pre”) were measured between 2.8 and 11.4 years pre-seroconversion; since the decline in the average TREC content in healthy individuals is negligible in such a period of time, we used these measurements as the best estimate of the TREC content at the moment of seroconversion. Cross-sectional data were collected during acute (n = 7) and chronic HIV infection (n = 27) as well as during progression to AIDS (n = 16) and were compared to age-matched healthy controls (n = 38). The p-values for the declines in phase I and phase II are given in the figure, while the difference between cross-sectional data from healthy and HIV-infected subjects are marked by an asterisk if the p-value<0.05.

Longitudinal analysis of CD4^+^ and CD8^+^ T-cell TREC contents revealed a biphasic decline during HIV-disease progression (Fig 1 and S1 Fig with true time since seroconversion). The average TREC contents showed a rapid and significant decline over seroconversion (average decline 0.60 log_10_/year for CD4^+^ and 1.02 log_10_/year for CD8^+^; slopes computed from the data of Fig 1), and hardly changed during the second phase of infection (Fig 1a and 1b). Notably, when analyzed cross-sectionally, neither the CD4^+^ T-cell TREC content 1 or 5 years after seroconversion, nor the CD4^+^ T-cell TREC content of additionally measured chronic typically progressing HIV-infected individuals (n = 27), differed significantly from those of 38 healthy age-matched controls (Fig 1a). Only HIV-infected individual who had progressed to AIDS (n = 16) had significantly lower TREC contents compared to healthy individuals (Fig 1a, p-value < 10^−4^), underlining the importance of longitudinal follow-up in TREC analyses. In contrast, TREC changes in the CD8^+^ T-cell pool could also be observed cross-sectionally (Fig 1b).

**Fig 2:**
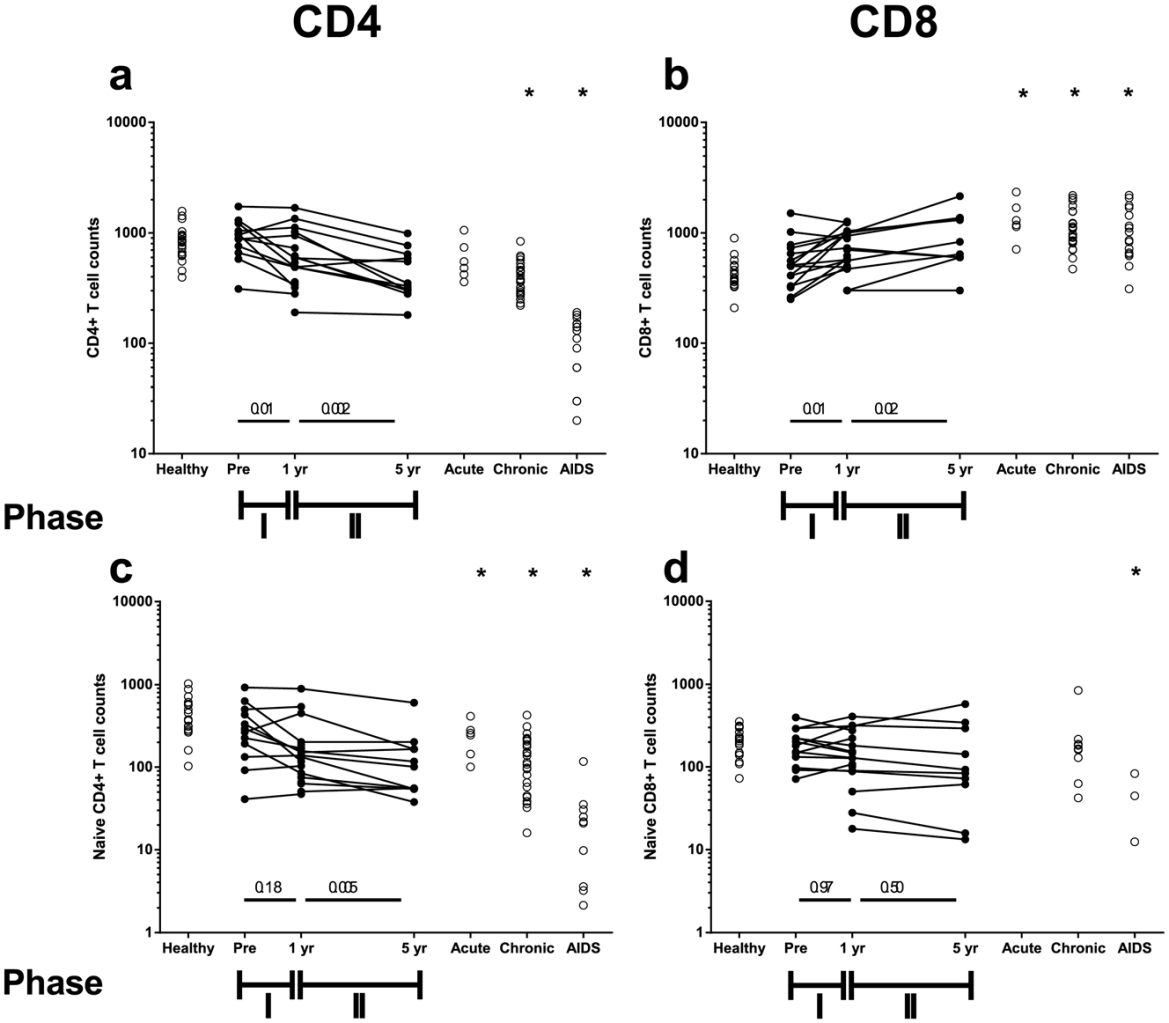
Total and naive CD4^+^ and CD8^+^ T-cell dynamics over seroconversion and during HIV infection. Total CD4^+^ T-cell counts (a), total CD8^+^ T-cell counts (b), naive CD4^+^ T-cell counts (c) and naive CD8^+^ T-cell counts (d) per μl of blood over seroconversion and during untreated HIV infection. Longitudinal data are connected by straight lines (n = 18), while cross-sectional data are denoted by open circles. The pre-seroconversion measurements (“Pre”) were measured between 2.8 and 11.4 years pre-seroconversion; since the change in T-cell numbers in healthy individuals is negligible in such a period of time, we used these measurements as the best estimate of the T-cell numbers at the moment of seroconversion. Cross-sectional data were collected during acute (n = 7) and chronic HIV infection (n = 27) as well as during progression to AIDS (n = 16) and were compared to age-matched healthy controls (n = 38). The p-values for the declines in phase I and phase II are given in the figure, while the difference between cross-sectional data from healthy and HIV-infected subjects are marked by an asterisk if the p-value<0.05.

In addition to TRECS, we longitudinally measured the percentages of CD4^+^ and CD8^+^ cells in the T-cell pool, as well as the percentage of naive and memory cells in the CD4^+^ and CD8^+^ T-cell pools during untreated HIV-1 infection. For CD4^+^ T cells, naive percentages were constant (S2a Fig), while total (Fig 2a), naive (Fig 2c) and memory (S2b Fig) CD4^+^ T-cell counts per μl of blood decreased significantly during phase II (for naive CD4^+^ average decline of 10 cells/μl/year corresponding to a decline of 1.3-fold between phase I and phase II). For CD8^+^ T cells, we found that naive percentages decreased significantly during phase I (S2a Fig), while total and memory CD8^+^ T-cell counts per μl of blood increased (Fig 2b and S1b Fig), and naive CD8^+^ T-cell counts per μl of blood were stable throughout HIV infection (Fig 2d).

As the average TREC content of memory T cells is much smaller than that of naive T cells [12,25], we estimated the average TREC content of naive CD4^+^ and CD8^+^ T cells by dividing the average TREC content of the CD4^+^ and CD8^+^ T-cell populations by the fraction of naive cells in the respective populations (S2 Fig), as previously suggested [25]. After this correction for the observed changes in the naive-memory ratio during HIV infection, TREC content changes in both CD4^+^ and CD8^+^ naive T cells remained biphasic, with a significant loss during phase I, and no further loss during phase II for both T-cell populations (Fig 1c and 1d).

### Increased naive T-cell division and loss rates suffice to explain the dynamics of naive T-cell numbers and TREC contents during HIV-1 infection

Our recent deuterium labeling studies have pointed out that naive and memory CD4^+^ and CD8^+^ T-cell dynamics are at least three-fold faster during chronic (untreated) HIV-1 [17]. Here, we investigated whether these changes in T-cell dynamics suffice to explain the observed biphasic loss of TRECs in naive T cells concomitant with a continual loss of naive CD4^+^ T cells and stable naive CD8^+^ T-cell numbers during HIV-1 infection.

We used a previously published mathematical model [9] describing the dynamics of naive T-cell numbers and their TREC over time. In this model, naive T cells are produced by the thymus in a time-dependent manner *σ(t)* and by peripheral T-cell division at a rate *p* and are lost at a rate *d* (see Supplemental Methods in S1 File). Using this model, we predicted the dynamics of naive CD4^+^ and CD8^+^ T-cell numbers and their TRECs in HIV-1 infection by i) increasing the naive T-cell loss rates *d* in accordance with the turnover estimates of our deuterium labeling studies among chronic HIV-1 infected individuals [17], and ii) deducing the peripheral T-cell division rates of naive CD4^+^ and CD8^+^ T cells from the combination of *d* and the observed net loss of these cells in HIV infection (see Supplemental Methods in S1 File).

**Fig 3:**
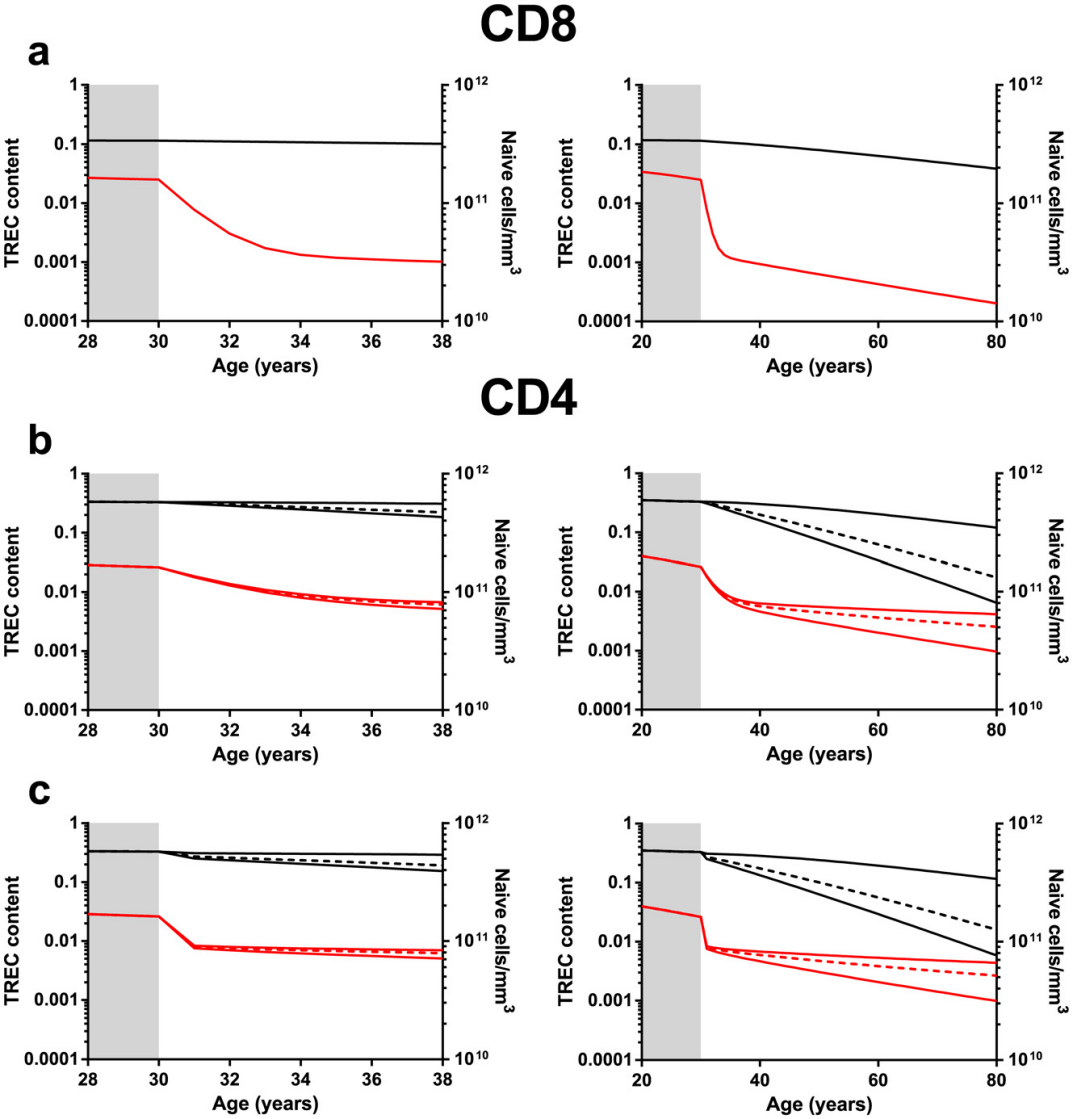
Predicted naive T-cell and TREC-content dynamics if HIV increases T-cell division and loss rates. Simulation results of naive T-cell counts (in black) and average TREC contents (in red) for an individual infected by HIV at the age of 30, assuming that the naive T-cell division rate increased according to the constraints described in Supplemental Methods. Left panels show the short-term dynamics while right panels show the long-term dynamics. Panel a shows the results for CD8^+^ T cells, Panels b and c for CD4^+^ T cells. In panel c, we have modeled the effect of increased turnover during the acute phase of infection, by using a 5-fold higher value of both *d* and *p* during the first 6 months of infection compared to the later stage of infection. For CD4^+^ T cells (panels b and c) we plotted the upper and lower bounds on the parameter constraints (denoted by the solid lines (*k*_*p*_ = *k*_*d*_ and *k*_*p*_ = *d(k*_*d*_*—1)/p+1*, see Supplemental Methods) as well as an intermediate case (denoted by the dashed lines (*k*_*d*_<*k*_*p*_< *d(k*_*d*_*—1)/p+1*) assuming that the thymic output and the average number of TREC per RTE are not changed after HIV infection (i.e. *k*_*s*_ = 1 and *k*_c_ = 1). Parameters: Panel a: before infection *σ*_*0*_ = 1.09×10^10^ cells/year, *h* = 1.5×10^11^cells, *d* = 0.109/year, *c* = 0.25 and *ν* = 0.05/year; after infection: *d* = 1.314/year and *p* = 1.300/year. Panels b and c: before infection *σ*_*0*_ = 3.65×10^10^ cells/year, *h* = 3.2×10^11^, *d* = 0.183/year, *c* = 0.25 and *ν* = 0.05/year; after infection: *d* = 0.548/year, *p* = 0.498, 0.510 or 0.532/year. Panel c: *d* = 2.740/year, *p* = 2.490, 2.550 or 2.660/year during the first six months of infection.

To account for constant naive CD8^+^ T-cell numbers during the first 5 years of HIV infection, the observed increase in the naive CD8^+^ T-cell loss rate [17] should go hand in hand with an even stronger increase in the peripheral CD8^+^ T-cell division rate (see Supplemental Methods in S1 File). We first tested the very conservative assumption that thymic output is not affected by HIV-1 infection (i.e. similar to an age-matched healthy individual), these parameter changes turned out to be sufficient to mimic the dynamics of naive CD8^+^ T-cell numbers and their average TREC contents that were observed experimentally (Fig 3a). Both the approximately 10-fold reduction in the average TREC content of naive CD8^+^ T cells and the biphasic nature of this decline thus seem natural consequences of the changes in T-cell loss and division rates upon HIV infection. Because some studies have suggested that thymic output is reduced by HIV-1 infection [8,10], we also predicted how an additional reduction of thymic output would affect the dynamic changes in the average CD8^+^ T-cell TREC content. Our simulations pointed out that thymic output reduction does not help to explain the biphasic nature of the CD8^+^ T-cell TREC decline in the first years of HIV infection (S3a Fig). If thymic output is reduced, the second stable phase of the average CD8^+^ T-cell TREC content is expected to be reached later and at a lower level, or alternatively, the decline may even become mono-phasic in the case of total loss of thymic output (S3a Fig).

For CD4^+^ T cells the situation is more challenging, as declining TREC contents concur with declining cell numbers. Basically, this puts two constraints on the rate of peripheral T-cell division: *p* should remain small enough to ensure a naive T-cell loss, and large enough to ensure a naive CD4^+^ T-cell TREC decline (see Supplemental Methods in S1 file). Within these constraints, our simulations indeed showed a biphasic decrease in the average TREC content and a loss of naive T cells, but the time scale of the biphasic behavior is much slower than experimentally observed (Fig 3b). Thus, the increases in T-cell turnover as measured by deuterium labeling in chronic HIV-1 infected individuals [17] were insufficient to explain the short- and long-term dynamics of both TREC contents and naive T-cell numbers in the CD4^+^ T-cell pool during HIV infection. Also reduced or abolished thymic output could not explain the observed loss of TRECs in the CD4^+^ T-cell pool during the first years of HIV infection (S3b Fig).

An alternative possibility is that the altered T-cell dynamics measured during chronic HIV-1 infection [17] are not representative for the changes occurring during acute infection. Indeed, expression of the cell division marker Ki67 in T cells has been reported to be higher during acute infection than during the chronic phase [19,26–28]. We studied if an even higher increase in T-cell loss and division rates during the early stage of infection could explain the observed biphasic decline of the average TREC content and the decline in naive CD4^+^ T-cell counts in HIV-1 infection. Indeed, by increasing the division and loss rates of naive CD4^+^ T cells 5-fold more during the first six months of HIV infection than measured during chronic infection (Table 1), our simulations yielded the fast initial TREC decline that we observed experimentally (Fig 3c). This fast initial decline was followed by a stable phase of the TREC content, and a fair decline of naive CD4^+^ T-cell numbers. Of note, adding such a temporary stronger increase in T-cell turnover for naive CD8^+^ T cells yielded an even larger and faster initial decline in TRECs for the CD8^+^ T-cell pool (S4 Fig).

**Table 1:** Parameter values used to describe the average TREC content and naive T-cell dynamics during HIV infection under the conservative assumption that thymic output and the average TREC content of recent thymic emigrants is not affected.

Parameter (/year)	CD8^+^			CD4^+^		
	healthy	acute	chronic	healthy	acute	chronic
Loss rate (*d*)	0.109	1.314	1.314	0.183	2.740	0.548
Division rate (*p*)	0.101^a^	1.300	1.300	0.166^a^	2.490–2.660^b^	0.498–0.532^b^

^a^ at 30 years of age

^b^ interval of possible values of the naive CD4^+^ T cells division rate to ensure a decline in both naive T-cell counts and their average TREC content (see Supplemental Methods in S1 File)

In summary, the observed changes in naive T-cell numbers and their TREC contents in the CD8^+^ T-cell pool can be explained by the observed increases in peripheral T-cell division and loss in HIV-1 infection. The short-term and long-term dynamics of naive T-cell numbers and their TREC contents in the CD4^+^ T-cell pool can also be explained by changes in T-cell division and loss rates, but only if these rates are more increased during acute than during chronic infection. Although we cannot exclude the possibility that thymic output may be affected by HIV-1, a reduction of thymic output does not help to resolve the longitudinal dynamics of TRECs and naive T cell numbers during HIV-1 infection.

## Discussion

Our longitudinal analysis of the naive CD4^+^ and CD8^+^ T-cell pools − over seroconversion and up to the chronic stage of HIV infection − shows that both CD4^+^ and CD8^+^ T-cell TREC dynamics are biphasic, with a rapid decline during the first year and a slow decline during the chronic phase of HIV infection. Although decreased CD4^+^ and CD8^+^ T-cell TREC contents have repeatedly been reported in HIV-1 infection [9,10,12,13], results from cross-sectional studies are conflicting. In one study, no significant decrease in CD4^+^ T-cell TREC contents could be detected during acute HIV infection [19], while in another the average TREC content of PBMC was found to be reduced in only half of the HIV-infected individuals [12]. Yet another cross-sectional study reported that HIV-infected individuals with high CD4^+^ T-cell counts had higher CD4^+^ T-cell TREC contents, while those with low CD4^+^ T-cell counts had lower CD4^+^ T-cell TREC contents than age-matched healthy controls [18]. The current longitudinal study demonstrates that both CD4^+^ and CD8^+^ TREC changes do occur rapidly upon HIV infection; because of large inter-individual differences in TREC measurements, such TREC changes can easily go unnoticed in cross-sectional studies. This study thereby also illustrates the caution that should be taken when interpreting cross-sectional TREC data in a longitudinal manner.

Our longitudinal analysis revealed a decrease in both total and naive CD4^+^ T-cell counts during HIV-1 infection, in line with previous observations [6]. Conversely, total and memory CD8^+^ T-cell counts increased, while naive CD8^+^ T-cell numbers remained relatively stable during the first five years of infection. In an early cross-sectional study, Roederer et al. [29] reported that both naive CD4^+^ and naive CD8^+^ T cells were depleted in HIV-1 infected individuals, especially in those with low CD4^+^ T-cell counts. The fact that the patients in our study still had relatively high CD4^+^ T-cell counts may explain why we did not observe such reduced naive CD8^+^ T-cell counts.

Using a mathematical model, we found that both the short-term and the long-term changes in TRECs and naive T-cell numbers during HIV infection may be a direct result of increased peripheral naive T-cell division and loss. Especially for CD8^+^ T cells, a biphasic loss of TRECs concomitant with relatively stable naive T-cell numbers can be explained by the observed increases in T-cell turnover [17], provided that the division rate of naive CD8^+^ T cells is increased more than their loss rate. In order to explain the observed dynamics of TRECs and naive T-cell numbers in the CD4^+^ T-cell pool, on the other hand, the turnover of naive T cells during the acute phase of infection should have been higher than we observed using deuterium labeling during chronic infection. There are indeed experimental data supporting that acute HIV infection is associated with higher levels of naive T-cell activation [26,27,30]. The early phase of infection is characterized by increased interleukin (IL)-4 and IL-10 production and alterations in the expression of phenotypic markers, which closely resembles the more advanced phases of HIV infection [27]. These immunologic alterations are thought to be transient and are followed by a return to a more normal profile [27].

Although our mathematical model successfully mimics the experimental observations, the required increases in the naive T-cell division rates are surprisingly restricted. The increase needs to be large enough to obtain a TREC decline, but small enough to avoid an increase in the number of naive T cells during HIV infection. Remarkably, even if thymus output were to be completely lost during HIV infection, the range of naive T-cell division rates for which these two requirements are fulfilled remains extremely small (see Supplemental Methods in S1 File). These constraints on naive T-cell division and loss have not always been obeyed in models for HIV infection [9], and suggest that the increases in T-cell division and death in HIV-1 infection are intrinsically linked.

The fact that we can mimic the experimental data by purely changing T-cell division and loss rates shows that the observed initial rapid TREC loss is not necessarily due to a rapid loss of a population of short-lived naive T cells, e.g. those that recently emigrated from the thymus. It is commonly thought that the naive T-cell pool contains a sub-population of relatively short-lived recent thymic emigrants (RTE) and it has been suggested that this population may be the one that is quickly lost after HIV infection, thereby explaining both changes in the TREC content and naive T-cell declines. However, our *in vivo* labeling studies in healthy individuals [16,17] have shown that naive T-cells: (i) are very long-lived, (ii) are mostly maintained by peripheral proliferation while thymic output contributes for only up to 10% of newly formed naive T-cells in a healthy adult, and (iii) do not present any signs of kinetic heterogeneity, suggesting that either all naive T-cells have the same turnover or that RTE are a negligible sub-population of naive T-cells. Therefore, we do not believe that the loss of RTE is a plausible alternative explanation for the TREC content and naive T-cell decline. This does not exclude the possibility that thymic output may nevertheless be reduced in HIV infection. Indeed, it has previously been shown that the average Sj/Vβ TREC ratio of PBMC declines within the first three months of HIV infection, suggesting that HIV reduces intra-thymic T-cell proliferation [8] and therefore would lead to an increased TREC content of recent thymic emigrants (*c* in our model). Assuming an increased value of *c* in HIV-infected individuals would yield even more restrictive constraints on the parameters (see Supplemental Methods in S1 File). Of note, as the contribution of thymic output to the maintenance of the naive T-cell pool in young healthy adults is small [16], a loss of thymic output − on top of the dynamic changes in T-cell turnover rates that we estimated [17] − is not expected to have a major impact on the peripheral naive T-cell pool of HIV-1 infected individuals (S2 Fig). Likewise, we cannot exclude that factors such as T-cell redistribution from the blood to the lymphoid organs during HIV-1 infection may play a role [31]. Whether or not such changes occur, we here show that even the mere dynamic changes in T-cell turnover suffice to explain the changes in the naive T-cell pool of HIV-1 infected individuals.

## Supporting Information

S1 Fig Average CD4+ and CD8+ TREC content changes after HIV infection. (PDF)

S2 Fig Changes in CD4^+^ and CD8^+^ T-cell subsets over seroconversion and during HIV infection. (PDF)

S3 Fig Predicted changes in naive CD4^+^ and CD8^+^ T-cell numbers and TREC contents if HIV also reduces thymic output. (PDF)

S4 Fig Predicted CD8^+^ naive T-cell and TREC-content dynamics if HIV increases T-cell division and loss rates with acute changes. (PDF)

S1 File Supplemental Methods. (PDF)
