# Supplementary material for: Reconciling Longitudinal Naive T-Cell and TREC Dynamics during HIV-1 Infection
Source: PLoS One. 2016 Mar 24;11(3):e0152513. doi: 10.1371/journal.pone.0152513 (PMC4806918; doi:10.1371/journal.pone.0152513)
Supplement: S1 Fig — (PDF) [file pone.0152513.s001.pdf]

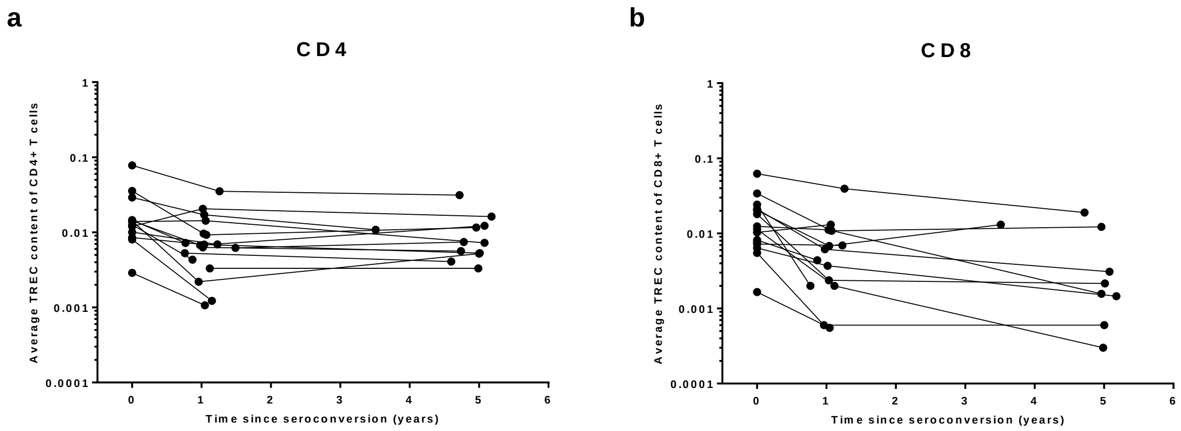

<u>Supplemental Figure S1</u>: Average CD4+ and CD8+ TREC content changes after HIV infection. Average CD4+ and CD8+ TREC contents measured since seroconversion. Average $CD4^+$ TREC contents (a) and average $CD8^+$ TREC contents (b) measured over seroconversion (time = 0) and during HIV infection. Longitudinal data are connected by straight lines.
