# Supplementary material for: Reconciling Longitudinal Naive T-Cell and TREC Dynamics during HIV-1 Infection
Source: PLoS One. 2016 Mar 24;11(3):e0152513. doi: 10.1371/journal.pone.0152513 (PMC4806918; doi:10.1371/journal.pone.0152513)
Supplement: S2 Fig — (PDF) [file pone.0152513.s002.pdf]

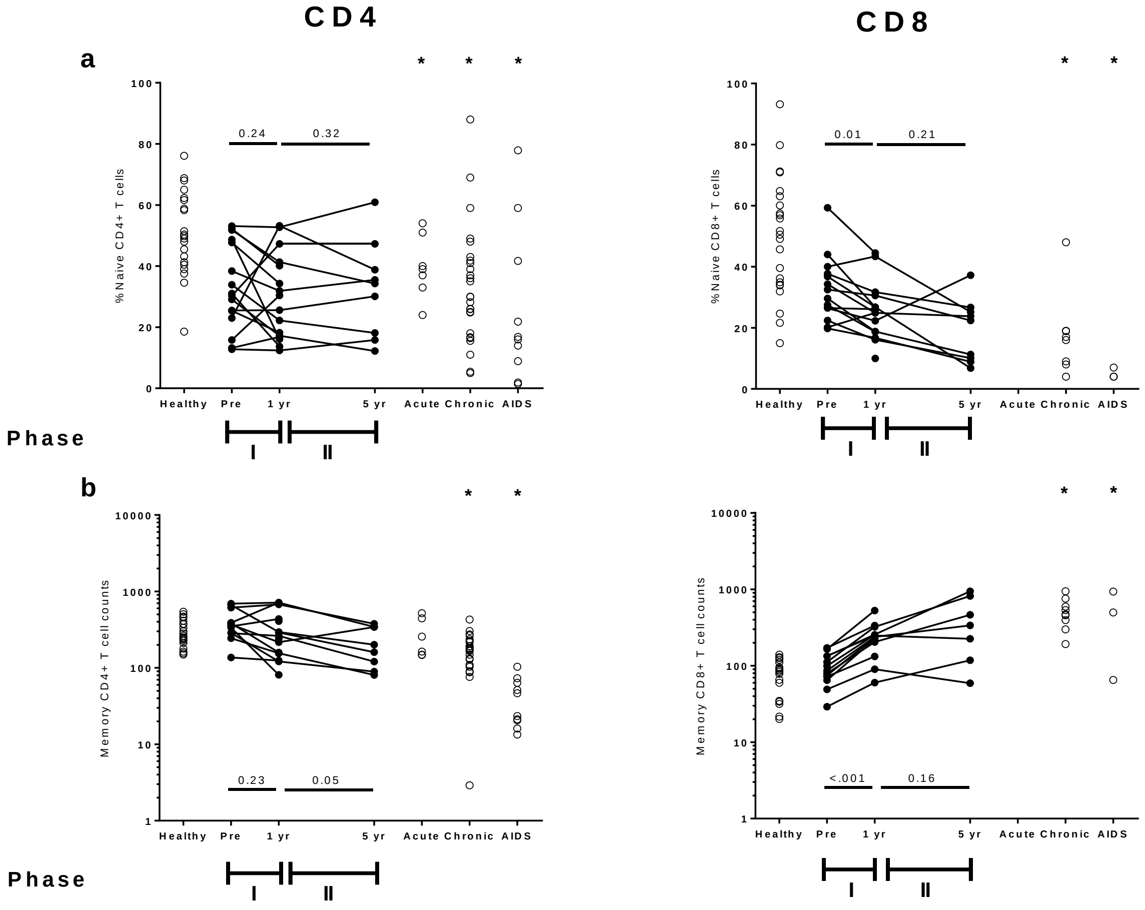

<u>Supplemental Figure S2</u>: Changes in CD4$^+$ and CD8$^+$ T-cell subsets over seroconversion and during HIV infection. Panel A gives the changes in the percentage of naive T cells, while panel B presents the dynamics of memory T-cell numbers, for CD4$^+$ (left panels) and CD8$^+$ (right panels) T cells. Longitudinal data are connected by straight lines, while cross-sectional data are denoted by open circles. The pre-seroconversion measurements ("Pre") were measured between 3-11 years pre-seroconversion, but since the loss in TRECs in healthy individuals is negligible in those years we plotted it at the time of seroconversion. Cross-sectional data were collected during acute (n=7) and chronic HIV infection (n=27) as well as during progression to AIDS (n=16) and were compared to age-matched healthy controls (n=38). P-values for phase I and phase II are indicated in the figure, and p-values for the difference between cross-sectional data from healthy and HIV-infected subjects are marked by an asterisk if $p<0.05$.
