# Supplementary material for: Reconciling Longitudinal Naive T-Cell and TREC Dynamics during HIV-1 Infection
Source: PLoS One. 2016 Mar 24;11(3):e0152513. doi: 10.1371/journal.pone.0152513 (PMC4806918; doi:10.1371/journal.pone.0152513)
Supplement: S3 Fig — (PDF) [file pone.0152513.s003.pdf]

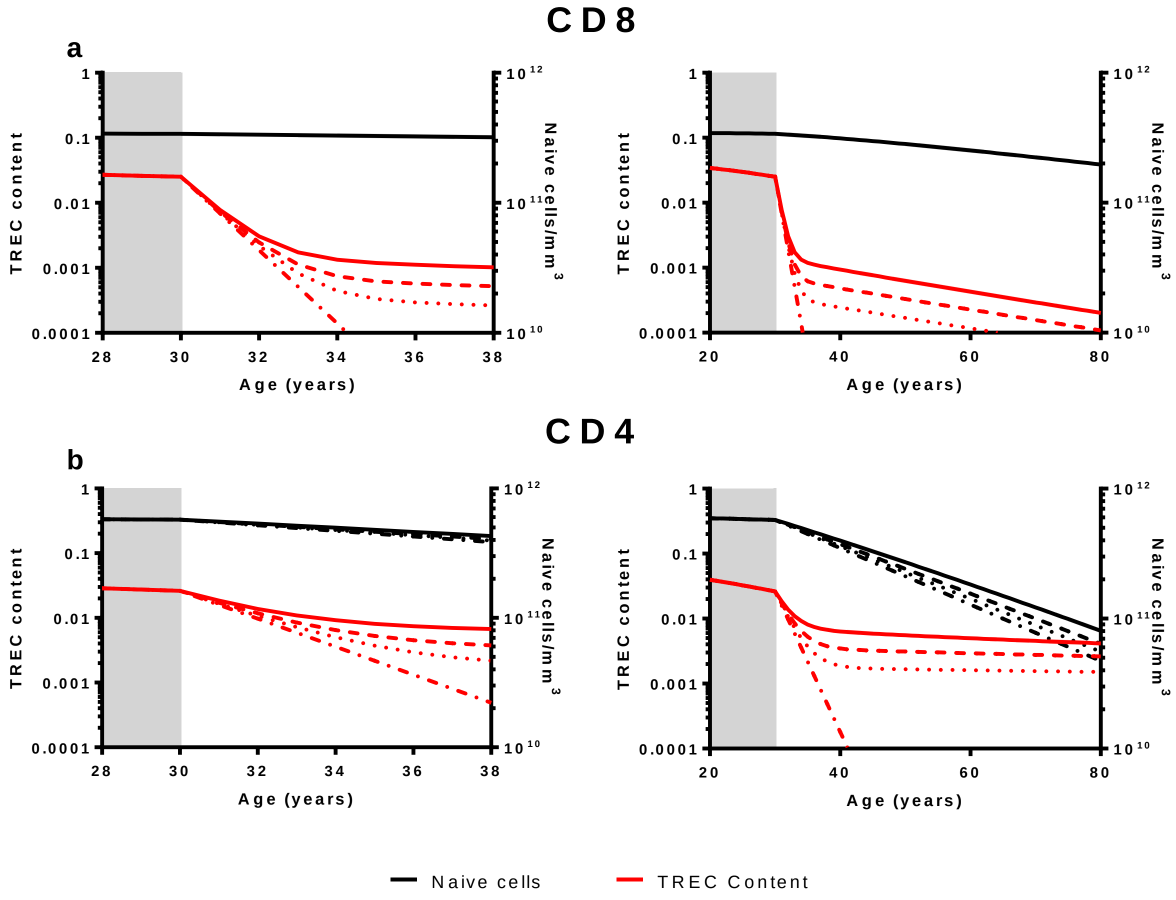

<u>Supplemental Figure S3</u>: Predicted changes in naive CD4$^+$ and CD8$^+$ T-cell numbers and TREC contents if HIV also reduces thymic output. The predicted effect of a reduction in thymic output on naive T-cell numbers (black curves) and their average TREC contents (red curves) if T-cell division and loss rates are increased as described in Figs. 3a and 3b. The solid lines represent the dynamics when thymic output is not affected by HIV (i.e. k_s=1), the dashed and dotted lines represent simulations in which thymic output was reduced to 50% and 25% of that in age-matched healthy controls (i.e. k_s=0.50 and 0.25), respectively, while the dotted-dashed line represents simulations in which thymic output was totally lost upon HIV infection (i.e. k_s=0). Left panels show the short-term dynamics while right panels show the long-term dynamics. Panel a: before infection $\sigma_0=1.09\times10^{10}$ cells/year, $h=1.5\times10^{11}$, c=0.25, ν=0.05/year and d=0.109/year. After infection: d=1.314/year and p=1.304/year, p=1.306/year and p=1.308/year for k_s=0.50, 0.25 and 0 respectively. Panel b: before infection $\sigma_0=3.65\times10^{10}$ cells/year, $h=3.2\times10^{11}$, c=0.25, ν=0.05/year and d=0.183/year. After infection: d=0.548/year and p=0.498/year (assuming the lowest possible increase of division rate, see Supplemental Methods).
