# Supplementary material for: Reconciling Longitudinal Naive T-Cell and TREC Dynamics during HIV-1 Infection
Source: PLoS One. 2016 Mar 24;11(3):e0152513. doi: 10.1371/journal.pone.0152513 (PMC4806918; doi:10.1371/journal.pone.0152513)
Supplement: S4 Fig — (PDF) [file pone.0152513.s004.pdf]

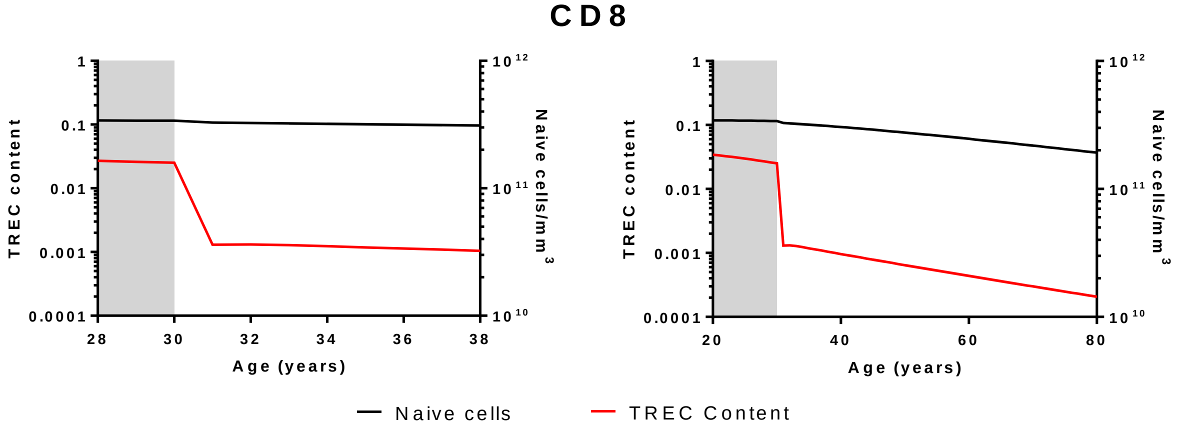

<u>Supplemental Figure S4</u>: Predicted CD8$^+$ naive T-cell and TREC-content dynamics if HIV increases T-cell division and loss rates with acute changes. Simulation results of naive T-cell counts (in black) and average TREC contents (in red) for an individual infected by HIV at the age of 30, assuming that the naive T-cell division rate increased according to the constraint described in Supplemental Methods. The left panel shows the short-term dynamics while the right panel shows the long-term dynamics. <u>Parameters</u>: before infection σ_0=1.09×10^{10} cells/year, h=1.5×10^{11}cells, d=0.109/year, c=0.25 and v=0.05/year; after infection: d=1.314/year and p=1.300/year. Both d and p are 5-fold higher during the first 6 months of infection.
