# Supplementary material for: Reconciling Longitudinal Naive T-Cell and TREC Dynamics during HIV-1 Infection
Source: PLoS One. 2016 Mar 24;11(3):e0152513. doi: 10.1371/journal.pone.0152513 (PMC4806918; doi:10.1371/journal.pone.0152513)
Supplement: S1 File — (PDF) [file pone.0152513.s005.pdf]

Supplemental Methods

Mathematical modeling of naive T-cell and TREC dynamics

Naive T-cell and TREC dynamics were investigated using a previously developed mathematical model [1]. In this model, N is the total number of naive $CD4^+$ T cells and T the total amount of TRECs in the naive T-cell population, $\sigma(t)$ is the time-dependent source of naive T cells from the thymus where t is the age of the individual in years, c is the average number of TRECs per recent thymic emigrant, d the rate of naive T-cell loss through cell death and priming of naive into memory cells, and p the rate of naive T-cell division (all expressed per year). The model can be written as follows:

$$\frac{dT}{dt} = c\sigma(t) - dT$$

$$\frac{dN}{dt} = \sigma(t) + p(N)N - dN$$

where $\sigma(t) = \sigma_0 e^{-\upsilon t}$. Thymic output is assumed to decay exponentially at rate υ per year and σ_0 represents thymus production of naive T cells at birth. Since TRECs can only be produced in the thymus, the total number of TRECs is not affected by peripheral T-cell proliferation. The average TREC content of naive T cells is defined as $A=T/N$ and changes according to $dA/dt = \sigma(t)\,(c - A)/N - p(N)A$. Both the T-cell loss rate d and the division rate p may depend on cell densities. We let the model allow for a density-dependent T-cell division rate by defining $p(N) = e^{-N/h}$. The quasi-steady state number of naive T cells $\bar{N}$ is defined by $\bar{N} = \frac{\sigma(t)}{d-p(\bar{N})}$. The quasi steady state of TREC content is computed from setting dA/dt=0 and is $\bar{A} = \frac{c\,\sigma(t)}{\sigma(t)+p(\bar{N})\bar{N}}$.

We considered the dynamics of naive T-cell numbers and their average TREC contents in healthy individuals over age as a starting point to simulate their dynamics during HIV infection. Based on our previous study among healthy adults, we assumed that the production of naive T cells before HIV seroconversion was mainly due to peripheral T-cell division, and that the thymus produced maximally 10% of all newly generated naive T cells [2,3]. The rates of naive T-cell loss, d, were set to the average turnover estimates obtained from our deuterium labeling studies among healthy young men [4]. The involution rate of the thymus was fixed at 0.05/year according to a previous study [5,6], and c was fixed at 0.25 to be in agreement with the measured TREC content of single positive [3]. Assuming that at the age of 30, 90% of naive T cells were formed through peripheral T-cell division, we manually tuned the parameter h such that the average TREC content A divided by c (i.e. the relative contribution of thymic output in the total production of naive T-cells) at age 30 was 0.1 [3]. The actual values of p and d are provided in Table 1.

Simulating HIV infection

We simulated the effects of HIV infection (at the arbitrary age of 30) by increasing the naive T-cell loss rate, d, to the average naive T-cell turnover rates estimated from our deuterium labeling study among untreated chronically HIV-1 infected individuals [7]. We deduced the corresponding T-cell division rates after HIV infection from the observed net loss rates of naive T cells, as follows:

Let the new thymic output, the average number of TRECs per recent thymic emigrant, T-cell division rate and T-cell loss rate during HIV infection be $\sigma' = k_s\sigma$, $c' = k_c c$, $p' = k_p \bar{p}$ and $d' = k_d d$, where $\bar{p} = p(N_{30})$, and the k parameters denote the fold change of each parameter upon HIV infection. Note that the parameters k_c, k_p and k_d will be larger than 1 while k_s should be smaller than, or equal to, 1. To obtain a TREC content decline, naive T cells should make more divisions during their lifespan after HIV infection, meaning that k_p should be larger than k_d. This intuitive result can be deduced from the steady states of the TREC content before and after HIV infection ($\bar{A}_{\mathrm{HIV}} < \bar{A}_{\mathrm{healthy}}$) which after some algebra leads to the inequality:

$$\frac{k_s\sigma(k_c-1)}{p\,\bar{N}_{HIV}} + k_s k_c \frac{\bar{N}_{healthy}}{\bar{N}_{HIV}} < k_p.$$

Substituting the steady states of the number of naive T cells before ($\bar{N}_{\mathrm{healthy}}$) and after HIV infection ($\bar{N}_{\mathrm{HIV}}$) this gives the constraint $k_d + \frac{(d-p)(k_c-1)k_d}{pk_c} < k_p$.

A second constraint on k_p follows from the steady states of the numbers of naive T cells before and after HIV infection: $\bar{N}_{\mathrm{HIV}} < \bar{N}_{\mathrm{healthy}}$ for naive CD4$^+$ T cells, while $\bar{N}_{\mathrm{HIV}} = \bar{N}_{\mathrm{healthy}}$ for naive CD8$^+$ T cells (Fig. 2). For CD4$^+$ T cells, the inequality implies that $k_p < \frac{d}{\bar{p}}(k_d - k_s) + k_s$, thus the combined constraints on k_p for CD4$^+$ T cells are:

$$k_d + \frac{(d-p)(k_c-1)k_d}{pk_c} < k_p < \frac{d}{\bar{p}}(k_d - k_s) + k_s,$$

while for CD8$^+$ T cells:

$$k_d + \frac{(d-p)(k_c-1)k_d}{pk_c} < k_p = \frac{d}{\bar{p}}(k_d - k_s) + k_s.$$

Assuming that thymic output and the average TREC content in recent thymic emigrants are not affected by HIV infection (i.e. k_s=1 and k_c=1) and given that k_d=12 for CD8$^+$ and k_d=3 for CD4$^+$ naive T cells [7], we deduced that $p' = k_p p$=1.3/year for CD8$^+$ naive T cells, and 0.498 < p'< 0.532/year for CD4$^+$ naive T cells, respectively (see Table 1). If thymic output is reduced by HIV infection, the constraints on k_p for CD4$^+$ T cells get slightly more relaxed; for example, in the extreme case in which HIV infection would totally block thymic output (i.e. k_s=0), the constraints would be p'=1.308/year for CD8$^+$ naive T cells, and 0.498 < p'< 0.549/year for CD4$^+$ naive T cells. If the average TREC content in recent thymic emigrants is increased by HIV infection, the constraints on k_p get even more restrictive.

References

1. Hazenberg MD, Otto SA, Cohen Stuart JW, Verschuren MC, Borleffs JC, Boucher CA, Coutinho RA, Lange JM, Rinke de Wit TF, Tsegaye A, van Dongen JJ, Hamann D, de Boer RJ, Miedema F (2000) Increased cell division but not thymic dysfunction rapidly affects the T-cell receptor excision circle content of the naive T cell population in HIV-1 infection. Nat Med 6: 1036-1042.

2. Murray JM, Kaufmann GR, Hodgkin PD, Lewin SR, Kelleher AD, Davenport MP, Zaunders JJ (2003) Naive T cells are maintained by thymic output in early ages but by proliferation without phenotypic change after age twenty. Immunol Cell Biol 81: 487-495.

3. den Braber I, Mugwagwa T, Vrisekoop N, Westera L, Mogling R, de Boer AB, Willems N, Schrijver EH, Spierenburg G, Gaiser K, Mul E, Otto SA, Ruiter AF, Ackermans MT, Miedema F, Borghans JA, de Boer RJ, Tesselaar K (2012) Maintenance of peripheral naive T cells is sustained by thymus output in mice but not humans. Immunity 36: 288-297.

4. Vrisekoop N, den Braber I, de Boer AB, Ruiter AF, Ackermans MT, van der Crabben SN, Schrijver EH, Spierenburg G, Sauerwein HP, Hazenberg MD, de Boer RJ, Miedema F, Borghans JA, Tesselaar K (2008) Sparse production but preferential incorporation of recently produced naive T cells in the human peripheral pool. Proc Natl Acad Sci U S A 105: 6115-6120.

5. Steinmann GG, Klaus B, Muller-Hermelink HK (1985) The involution of the ageing human thymic epithelium is independent of puberty. A morphometric study. Scand J Immunol 22: 563-575.

6. Westera L, van Hoeven V, Drylewicz J, Spierenburg G, van Velzen JF, de Boer RJ, Tesselaar K, Borghans JA (2015) Lymphocyte maintenance during healthy aging requires no substantial alterations in cellular turnover. Aging Cell 14: 219-227.

7. Vrisekoop N, Drylewicz J, Van GR, Mugwagwa T, Van LS, Veel E, Otto SA, Ackermans MT, Vermeulen JN, Huidekoper HH, Prins JM, Miedema F, De Boer RJ, Tesselaar K, Borghans JA (2015) Quantification of naive and memory T-cell turnover during HIV-1 infection. AIDS 29: 2071-2080.
